# Nasal and perirectal colonization of vancomycin sensitive and resistant enterococci in patients of paediatrics ICU (PICU) of tertiary health care facilities

**DOI:** 10.1186/1471-2334-13-156

**Published:** 2013-03-27

**Authors:** Muhammad Arfat Yameen, Saira Iram, Abdul Mannan, Shujaat Ali Khan, Naeem Akhtar

**Affiliations:** 1 Department of Pharmaceutical Sciences, COMSATS Institute of Information Technology, Abbottabad 22060, Pakistan; 2 Microbiology Research Laboratory, Department of Microbiology, Quaid-I-Azam University, Islamabad 45320, Pakistan; 3 Department of Pathology, Rawalpindi Medical College, Rawalpindi, 46300, Pakistan; 4 Department of Microbiology, University of Health Sciences, Lahore, 54600, Pakistan

**Keywords:** Enterococci colonization, Antimicrobial susceptibility, Vancomycin resistant enterococci

## Abstract

**Background:**

Enterococci normally inhabit the intestinal tract of humans and are also a potential pathogen in causing nosocomial infections. The increase in antibiotic resistance and transfer of antibiotic resistance gene to *Staphylococcus aureus* (*S. aureus*) due to co-colonization has increased its importance in research. The aim of the study was to evaluate local epidemiology of nasal and rectal colonization with *Enterococcus faecalis* (*E. faecalis*) and *Enterococcus faecium* (*E. faecium*) in patients of Paediatrics Intensive Care Unit (PICU) and correlation with clinical and socioeconomic factors.

**Methods:**

The nasal and perirectal swab samples were collected from 110 patients admitted in PICUs of three tertiary care hospitals of Rawalpindi Medical College, Pakistan. The identification of enterococci was done by biochemical tests and by PCR for ddl, vanA and vanB genes. Antibiotic susceptibility testing was performed by disc diffusion and MICs were determined for vancomycin, tetracycline, ciprofloxacin and oxacillin only.

**Results:**

Out of 220 nasal and perirectal samples, 09 vancomycin-resistant enterococci (VRE) and 76 vancomycin-susceptible enterococci (VSE), consisting of 40 *E. faecalis* and 45 *E. faecium* were isolated. PCR successfully identified both species with *ddl* primers and VRE with *vanA* primer. With disc diffusion method, all isolates were resistant to most of the antibiotics tested except linezolid, quinupristin/dalfopristin, teicoplanin and vancomycin. VRE showed resistance to teicoplanin and vancomycin both and none was resistant to linezolid and quinupristin/dalfopristin. Generally, *E. faecium* isolates were more resistant than *E. faecalis*. MICs of vancomycin for nasal and perirectal VRE were 512 mg/L and 64 to 512 mg/L respectively. VRE were more in patients with prolonged hospitalization, from urban localities and those having pneumonia.

**Conclusion:**

Present study reveals high colonization and antibiotic resistance in enterococcal isolates from nasal and perirectal area. Nasal colonization by enterococci in PICU is more alarming as VRE may cause infection and can transfer this resistance gene to other microorganisms like *S. aureus*.

## Background

Enterococci normally inhabit the intestinal tract of humans and animals and may colonize the human oral cavity, vagina, hepatobiliary tract and skin of healthy individuals [1]. Two species, *E. faecalis* and *E. faecium*, have been more frequently isolated from clinical samples than other species of enterococci, accounting for 80 to 90% and 5 to 10%, respectively [2].

As compared to other Gram-positive organisms, enterococci have relatively low virulence but, in recent years they have emerged as the nosocomial pathogens of the 1990s [3-6]. Several factors, including ubiquitous distribution as intestinal flora and the widespread use of broad-spectrum antibiotics and invasive devices have contributed to the emergence of enterococci as important pathogens [7] and perhaps most important is their extensive resistance to a wide range of antimicrobial agents. These properties allow this organism to survive and multiply with a selective advantage over other fecal flora in a hospital environment where antimicrobial agents are heavily used. The aim of the study was to evaluate local epidemiology of nasal and rectal colonization with *E. faecalis* and *E. faecium* in PICUs patients and correlation with clinical and socioeconomic factors.

*S. aureus* persistently or intermittently can colonize the nasal cavity and perirectal area of healthy humans and transfer of vancomycin-resistant gene due to co-colonization with enterococci has been reported in some previous studies, so nasal samples were also collected with the hypothesis that enterococci (*E. faecalis* and *E. faecium*) can co-exist with *S. aureus* and may transfer resistance gene [8-11].

The study also included risk factors like age of the patient, reason for admission, stay of patient in the hospital at the time of sampling, residential (rural, urban) and socioeconomic status (high, middle, low) of the patients.

## Methods

The prospective microbiological surveillance study was carried out at Microbiology Laboratory, Holy Family Hospital, Rawalpindi, Pakistan and Microbiology Research Laboratory, Quaid-I-Azam University, Islamabad, Pakistan during the period from March to September 2010. After ethical approval of study, granted by Ethical Committee of RMC and Allied Hospitals, Rawalpindi, Pakistan (No. EC/1721-22/RMC/dated: 03/03/2010), written consents were taken from the parents and guardians of the children before sampling. Samples of the anterior nares and perirectal area were obtained from every patient admitted in PICUs of Allied Hospitals of Rawalpindi Medical College, Rawalpindi, Pakistan, either newly admitted or transferred from other units of the same or different hospitals. Patients with duplicate admissions during the study period were excluded.

Samples were processed within two hours of collection. The swabs were inoculated onto Bile Aesculin Agar (BAA) (Oxoid, UK) plates and were incubated at 45°C for 24 to 72 hours. Characteristic pinpoint colonies and colonies with black zone around were subcultured on Mueller Hinton Agar (MHA) with 6% NaCl (Oxoid, UK) at 45°C for confirmation. Further identification of these isolates was done by pink or red colonies on KF Streptococci Agar (KFSA) (Oxoid, UK), negative catalase and coagulase tests and gamma-hemolysis on Sheep Blood Agar (SBA) (Oxoid*,* UK) after overnight growth at 37°C. All confirmed enterococci isolates were preserved in 16% v/v glycerol broth and in Microbank tubes (Pro-Lab Diagnostics, US) at -70°C.

### Molecular identification

The species identification of enterococci (*E. faecalis* and *E. faecium)* was done using PCR by targeting ddl _*E. faecalis*_ and ddl _*E. faecium*_ genes. The vancomycin-resistant gene was identified with *vanA* and *vanB* primers. All the primer sequences used have been used in previous studies [12] and were obtained from Sigma Geno§ys (Sigma Aldrich, USA), Alpha DNA (Alpha DNA, Germany) and e-Oligo (Gene Link, USA) (Table 1).

**Table 1:** Oligonucleotides primers used for enterococci

**Primer designation**	**Sequences (5** → **3)**	**Product size (bp)**	**Company**	**Reference**
***E. faecium***				
*ddl*_*E. faecium*(1)_	TTGAGGCAGACCAGATTGACG	658	Alpha DNA/Sigma	Kariyama et al., 2000
*ddl*_*E*. *faecium*(2)_	TATGACAGCGACTCCGATTCC			
***E. faecalis***				
*ddl*_*E.faecalis*(1)_	ATCAAGTACAGTTAGTCT	941	Alpha DNA/Sigma	Kariyama et al., 2000
*ddl*_*E*. *faecalis*(2)_	ACGATTCAAAGCTAACTG			
*vanA *(1)	GGGAAAACGACAATTGC	732	Alpha DNA/e-Oligo	Kariyama et al., 2000
*vanA *(2)	GTACAATGCGGCCGTTA			
*vanB *(1)	GTGCTGCGAGATACCACAGA	635	Alpha DNA/e-Oligo	Kariyama et al., 2000
*vanB *(2)	CGAACACCATGCAACATTTC			

DNA was extracted with Wizard^®^ Genomic DNA purification kit (Promega Corporation, USA) according to manufacturer’s instructions. DNA was also extracted manually with Triton X lysis buffer by the method used in the previous study for DNA extraction from bacterial colonies [13]. The isolated DNA was stored at 2 to 8°C.

Amplification was carried out in Biometra T1 Thermocycler (Biometra, Germany) with initial denaturation at 95°C for 4 min, then 30 cycles of denaturation at 95°C for 30 sec., annealing at 52°C for 1 min and extension at 72°C for 2 min followed by final extension at 72°C for 7 min. The final PCR product was held at 4°C until removed.

The PCR product was analysed on 1% agarose gel. DNA ladder (O’Gene Ruler) of 100 bp and 1 kb was used to compare the size of PCR amplified fragments. Electrophoresis was done at 100 V for one hour and gel was viewed under Molecular Imager Gel Doc XR + System, Bio-Rad Laboratories, US.

### Antimicrobial susceptibility testing

Antimicrobial susceptibility testing was performed by Kirby-Bauer modified disc diffusion method [14] according to CLSI guidelines [15] using MHA plates. The antibiotic discs used were ampicillin, amoxicillin/clavulanic acid, methicillin, oxacillin, penicillin G, cephalexin, cefoxitin, cephalothin, cephradine, ciprofloxacin, levofloxacin, erythromycin, tetracycline, gentamicin, imipenem, linezolid, quinupristin/dalfopristin, teicoplanin and vancomycin. Minimum inhibitory concentrations (MICs) for ciprofloxacin, oxacillin, tetracycline and vancomycin were determined with agar dilution method according to British Society for Antimicrobial Chemotherapy Guidelines [16]. Standard antibiotic powders were obtained from MP Biomedicals UK. Stock solutions were prepared according to manufacturer’s instructions. Following breakpoint concentrations of these antibiotics were used: tetracycline 1 mg/L, oxacillin 2 mg/L, ciprofloxacin 4 mg/L and vancomycin 8 mg/L.

### Patient’s clinical data

Patient’s data, including age (<12 years), gender, residential and socioeconomic status, clinical diagnosis, history of vancomycin intake, surgical interventions, invasive procedure and devices, current medication profile was collected from the hospital record.

The Statistical Package for Social Sciences (SPSS) version 13.0 was used for statistical analysis by average, ±standard deviation, chi-square test (Cross tabulation) and *t*-test. The p-value ≤0.05 was considered as “statistical significant.”

## Results

In nasal samples, there were 29/110 (26.4%) enterococci, including 03/29 (10.3%) VRE (02 *E. faecalis* and 01 *E. faecium*) and 26/29 (89.7%) VSE (09 *E. faecalis* and 17 *E. faecium*)*.* The remaining 81/110 (73.6%) nasal samples gave no enterococcal growth (Table 2).

**Table 2:** Frequency of VRE and VSE in paediatrics intensive care units

	**VRE faecium**	**VRE faecalis**	**VSE faecium**	**VSE faecalis**	**No Enterococci**
	**n (%)**	**n (%)**	**n (%)**	**n (%)**	**n (%)**
**Nasal (n = 110)**	1 (0.9)	2 (1.8)	17 (15.5)	9 (8.2)	81 (73.6)
**Perirectal (n = 110)**	3 (2.7)	3 2.7)	24 (21.8)	26 (23.6)	54 (49.1)
**Total**	4	5	41	35	135

In perirectal samples, 56/110 (50.9%) enterococci were isolated with 06/56 (10.7%) VRE isolates (03 each of *E. faecalis* and *E. faecium*) and 50/56 (89.3%) VSE isolates (26 *E. faecalis* and 24 *E. faecium*). Fifty four perirectal samples gave no growth of enterococci (Table 2). Sixteen patients had enterococci in both their nasal and perirectal samples. Statistically significant association was found in nasal and perirectal enterococcal isolation (Chi-square test: 75.314, P < 0.001).

PCR successfully identified 85/220 (38.6%) enterococci in both nasal and perirectal samples with the help of *ddl*_*E. faecalis*_ and *ddl*_*E. faecium*_ primers. In these, 40/85 (47.1%) were *E. faecalis* and 45/85 (52.9%) were *E. faecium*. Similarly, all 09/85 (10.6%) VRE were identified with *vanA* primer. No enterococcal isolates gave amplification with *vanB* primer.

### Antimicrobial susceptibility testing

With disc diffusion method, all nasal and perirectal isolates were 66 to 100% resistant to cephalexin, cefoxitin, cephalothin, cephradine, ciprofloxacin, erythromycin, gentamicin, methicillin and oxacillin. Whereas nasal isolates were 36 to 55% resistant to penicillin G, ampicillin, amoxicillin/clavulanic acid, imipenem, levofloxacin and tetracycline, while perirectal isolates showed variable resistance to these antibiotics. Both nasal and perirectal isolates were 05 to 18% resistant to teicoplanin and vancomycin and were susceptible to linezolid and quinupristin/dalfopristin (Table 3). Perirectal *E. faecium* isolates showed higher resistance than *E. faecalis* in disc diffusion test.

**Table 3:** Antibiotics resistant pattern of nasal and perirectal enterococci by disc diffusion method

**Antibiotic discs (abbreviations)**	**Nasal isolates**		**Perirectal isolates**	
	**(n = 29)**		**(n = 56)**	
	**E. faecalis (n = 11)**	**E. faecium (n = 18)**	**E. faecalis (n = 29)**	**E. faecium (n = 27)**
**% Resistance**				
**Co-amoxyclav 30 μg **(AMC30)	36.4	50	13.8	70.4
**Ampicillin 25 μg **(AMP25)	36.4	50	13.8	70.4
**Cefoxitin 30 μg **(FOX30)	100	100	93.1	100
**Cephalexin 30 μg **(CL30)	90.9	77.8	100	96.3
**Cephalothin 30 μg **(KF30)	81.8	66.7	75.9	96.3
**Cephradine 30 μg **(CE30)	81.8	94.4	100	100
**Ciprofloxacin 5 μg **(CIP5)	81.8	66.7	69	96.3
**Erythromycin 15 μg **(E15)	90.9	88.9	79.3	96.3
**Gentamicin 30 μg **(CN30)	72.7	83.3	79.3	92.6
**Imipenem 10 μg **(IPM10)	45.5	55.6	24.1	85.2
**Levofloxacin 5 μg **(LEV5)	45.5	50	62.1	92.6
**Linezolid 30 μg **(LZD30)	0	0	0	0
**Methicillin 10 μg **(MET10)	100	100	96.6	100
**Oxacillin 1 μg **(OX1)	100	100	100	100
**Penicillin G 10 IU **(P10)	36.4	55.6	31	85.2
**Quinupristin/Dalfopristin 15 μg **(QD15)	0	0	0	0
**Teicoplanin 30 μg **(TEC30)	18.2	5.6	10.3	11.1
**Tetracycline 30 μg **(TE30)	36.4	50	75.9	70.4
**Vancomycin 30 μg **(VA30)	18.2	5.6	10.3	11.1

Among nasal isolates, 03/29 (10.3%) were VRE (break point (bp) ≥08 mg/L) and 26/29 (89.7%) were VSE (bp <08 mg/L). One isolate of *E. faecalis* and one isolate of *E. faecium* had MIC 512 mg/L (*t*-test, P > 0.05). MICs of tetracycline ranged from 02 to 256 mg/L (*t*-test, P < 0.05) for both nasal *E. faecalis* and *E. faecium* and all were tetracycline resistant (bp ≥01 mg/L). The MICs of ciprofloxacin for nasal *E. faecalis* and *E. faecium* ranged from 01 to 256 mg/L and 02 to 512 mg/L respectively. Only 04/29 (13.8%) isolates (02 from each species) were inhibited at concentration of <04 mg/L and were considered susceptible, rests of 25/29 (86.2%) were resistant. *T*-test statistic gave P > 0.05 for *E. faecalis* and P < 0.05 *E. faecium*. In case of oxacillin, MICs ranged from 08 to 512 mg/L (*t*-test, P < 0.05) and from 04 to 512 mg/L (*t*-test, P < 0.05) for *E. faecalis* and *E. faecium* respectively, indicating that all isolates are resistant (Table 4).

**Table 4:** MICs of nasal enterococci

	**Nasal isolates (n = 29)**							
	***E. faecalis *(n = 11)**				***E. faecium *(n = 18)**			
**Antibiotic dilutions**	**TET (%)**	**CIP (%)**	**OXA(%)**	**VAN (%)**	**TET (%)**	**CIP (%)**	**OXA (%)**	**VAN (%)**
**0.5**	----------	----------	----------	----------	----------	----------	----------	----------
**1**	^**†**^---------	1 (9.1)	----------	5 (45.5)	^**†**^---------	----------	----------	11 (61.1)
**2**	2 (18.2)	1 (9.1)	^**†**^---------	3 (27.3)	2 (11.1)	2 (11.1)	^**†**^---------	6 (33.3)
**4**	2 (18.2)	^**†**^---------	----------	1 (9.1)	1 (5.6)	^**†**^2 (11.1)	1 (5.6)	----------
**8**	1 (9.1)	3 (27.3)	1 (9.1)	^**†**^---------	3 (16.7)	2 (11.1)	1 (5.6)	^**†**^---------
**16**	----------	1 (9.1)	1 (9.1)	----------	1 (5.6)	----------	1 (5.6)	----------
**32**	----------	2 (18.2)	2 (18.2)	----------	----------	3 (16.7)	1 (5.6)	----------
**64**	----------	1 (9.1)	2 (18.2)	----------	1 (5.6)	----------	1 (5.6)	----------
**128**	3 (27.3)	1 (9.1)	2 (18.2)	----------	6 (33.3)	3 (16.7)	3 (16.7)	----------
**256**	3 (27.3)	1 (9.1)	1 (9.1)	----------	4 (22.2)	4 (22.2)	6 (33.3)	----------
**512**	----------	----------	2 (18.2)	2 (18.2)	----------	2 (11.1)	4 (22.2)	1 (5.6)
***t*-test**	P < 0.05	P = 0.076	P < 0.05	P = 0.195	P < 0.05	P < 0.05	P < 0.05	P = 454
	t = 3.182	t = 1.981	t = 2.772	t = 1.389	t = 4.488	t = 3.499	t = 5.239	t = 0.766

^† ^Breakpoint concentration, *TET*: Tetracycline, *CIP*: Ciprofloxacin, *OXA*: Oxacillin, *VAN*: Vancomycin.

In perirectal isolates, 06/56 (10.7%), 03 *E. faecalis* and 03 *E. faecium* were VRE with vancomycin MICs from 64 to 512 mg/L, while remaining 50/56 (89.3%), 26 *E. faecalis* and 24 *E. faecium* were VSE (*t*-test, P > 0.05). There is not much difference in MICs of perirectal isolates than those of nasal isolates. For tetracycline, 08/56 (14.3%) isolates, (05/29 *E. faecalis* and 03/27 *E. faecium*) were inhibited at 0.5 mg/L (bp >01 mg/L) and were susceptible, while remaining of the isolates were resistant showing MICs in range from 64 to 256 mg/L and 01 to 256 mg/L for both of species respectively (*t*-test, P < 0.05). For ciprofloxacin all *E. faecalis* and *E. faecium* were resistant with MICs from 04 to 512 mg/L (*t*-test, P < 0.05). The oxacillin MICs ranged from 8 to 512 mg/L for both isolates and were resistant (*t*-test, P < 0.05) (Table 5).

**Table 5:** MICs of perirectal enterococci

	**Perirectal isolates (n = 56)**							
	***E. faecalis *(n = 29)**				***E. faecium *(n = 27)**			
**Antibiotic dilutions**	**TET (%)**	**CIP (%)**	**OXA(%)**	**VAN (%)**	**TET (%)**	**CIP (%)**	**OXA(%)**	**VAN (%)**
**0.5**	5 (17.2)	----------	----------	----------	3 (11.1)	----------	----------	----------
**1**	^**†**^---------	----------	----------	12 (41.4)	^**†**^1 (3.7)	----------	----------	12 (44.4)
**2**	----------	1 (3.4)	^**†**^---------	12 (41.4)	----------	----------	^**†**^---------	10 (37)
**4**	----------	^**†**^4 (13.8)	----------	2 (6.9)	5 (18.5)	^**†**^2 (7.4)	----------	2 (7.4)
**8**	----------	5 (17.2)	1 (3.4)	^**†**^--------	----------	5 (18.5)	1 (3.7)	^**†**^--------
**16**	----------	2 (6.9)	7 (24.1)	---------	3 (11.1)	2 (7.4)	1 (3.7)	----------
**32**	----------	7(24.1)	8 (27.6)	---------	2 (7.4)	3 (11.1)	4 (14.8)	----------
**64**	2 (6.9)	----------	2 (6.9)	1 (3.4)	2 (7.4)	4 (14.8)	2 (7.4)	----------
**128**	10 (34.5)	3 (10.3)	2 (6.9)	---------	3 (11.1)	2 (7.4)	3 (11.1)	----------
**256**	12 (41.4)	5 (17.9)	3 (10.3)	---------	8 (29.6)	5 (18.5)	3 (11.1)	----------
**512**	----------	2 (6.9)	6 (20.7)	2 (6.9)	----------	4 (14.8)	13 (48.1)	3 (11.1)
***t*-test**	P < 0.05	P < 0.05	P < 0.05	P = 0.214	P < 0.05	P < 0.05	P < 0.05	P = 0.121
	t = 8.611	t = 3.666	t = 4.273	t = 1.271	t = 4.659	t = 4.204	t = 7.070	t = 1.601

^† ^Breakpoint concentration, *TET*: Tetracycline, *CIP*: Ciprofloxacin, *OXA*: Oxacillin, *VAN*: Vancomycin.

### Patient’s clinical data

In this study, the average patient stay was 5.42 (SD ± 5.79) days in PICU at the time of sampling. Statistically, no significant association was found in rate of enterococcal isolation with duration of stay of patients in PICU (Chi-square test: nasal: 20.505 & perirectal: 19.481, P > 0.05). However, the isolation of VRE both form nasal and perirectal area was more form patients who have longer hospital stay. There were 06/09 VRE isolates from patients who stayed more than two days in the unit (Table 6).

**Table 6:** Association of different risk factors with enterococci colonization

**Factors**	**No. of patients (n = 110)**	**VSE nasal enterococci (n = 26)**		**VSE perirectal enterococci (n = 50)**		**VRE nasal (n = 3)**	**VRE perirectal (n = 6)**
	**Number (%)**	***E. faecalis *(%)**	***E. faecium *(%)**	***E. faecalis *(%)**	***E. faecium *(%)**	***E. faecalis***	***E. faecalis***
						***E. faecium *(%)**	***E. faecium *(%)**
**Age of patients**							
· <1	69 (62.7)	8 (30.8)	12 (46.2)	17 (34)	14 (28)	1 (33.3)	2 (33.3)
· 1 to <6	29 (26.4)	0	5 (19.2)	7 (14)	7 (14)	1 (33.3)	3 (50)
· 6 to <12	12 (10.9)	1 (3.9)	0	2 (4)	3 (6)	1 (33.3)	1 (16.7)
**Reason for Admission**							
· Miscellaneous group	26 (23.6)	2 (7.7)	3 (11.5)	7(14)	9 (18)	1 (33.3)	1 (16.7)
· Aspiration Pneumonia	1 (0.9)	0	0	1 (2)	0	0	0
· Meningitis	11 (10)	0	1 (3.9)	3 (6)	1 (2)	0	2 (33.3)
· Pneumonia	68 (61.8)	7 (26.9)	13 (50)	15 (30)	14 (28)	2 (66.7)	3 (50)
· Renal Failure	2 (1.8)	0	0	0	0	0	0
· Tuberculosis	2 (1.8)	0	0	0	0	0	0
**Duration of stay**							
· 0 day	13 (11.8)	3 (11.5)	3 (11.5)	6 (12)	2 (4)	0	0
· 1 day	13 (11.8)	0	1 (3.9)	3 (6)	3 (6)	0	1 (16.7)
· 2 days	20 (18.2)	3 (11.5)	3 (11.5)	5 (10)	5 (10)	1 (33.3)	1 (16.7)
· 3 to 5 days	24 (21.8)	1 (3.9)	3 (11.5)	5 (10)	4 (8)	0	0
· 6 to 10 days	22 (20)	2 (7.7)	5 (19.2)	4 (8)	8 (16)	0	1 (16.7)
· >10 days	18 (16.4)	0	2 (7.7)	3 (6)	2 (4)	2 (66.7)	3 (50)
**Residential Status**							
· Rural	48(43.6)	2 (7.7)	6 (23.1)	9 (18)	11 (22)	0	1 (16.7)
· Urban	62 (56.4)	7 (26.9)	11 (42.3)	17 (34)	13 (26)	3 (100)	5 (83.3)
**Socioeconomic Status**							
· Lower Class	69 (62.7)	4 (15.4)	9 (34.6)	9 (18)	13 (26)	3 (100)	6 (100)
· Middle Class	40 (36.4)	5 (19.2)	8 (30.8)	16 (32)	11 (22)	0	0
· Higher Class	1 (0.9)	0	0	1 (2)	0	0	0
**Vancomycin use**							
· Yes	14 (12.7)	2 (7.7)	3 (11.5)	4 (8)	1 (2)	1(33.3)	3 (50)
· No	96 (87.3)	7 (26.9)	14 (53.8)	22 (44)	23 (46)	2 (66.7)	3 (50)

Patients with age group <1 year were more colonized with both types of enterococci than other age groups. These were 8/26 (30.8%) *E. faecalis* and 12/26 (34%) *E. faecium* from nasal (Chi-square test: 14.733, P > 0.05) and 17/50 (30.8%) *E. faecalis* and 14/50 (34%) *E. faecium* from perirectal samples (Chi-square test: 5.407, P > 0.05). The VRE isolation was almost equal in all the three age groups with no significant association with enterococci isolation.

All VRE isolates were from patients with lower socioeconomic class, while other classes were colonized with VSE only (Table 6). Socioeconomic status of patients showed significant association with respect to isolation of enterococci from perirectal area (Chi-square test: 19.163, P < 0.05), but no association with nasal isolates was found (Chi-square test: 4.598, P > 0.05). The patients on vancomycin treatment were colonized with 01/03 (33.3%) nasal and 03/06 (50%) perirectal VRE. There was significant relationship of vancomycin treatment with perirectal enterococcal isolation (Chi-square test: 10.881, P < 0.05) while no significant association was present with nasal isolates (Chi-square test: 4.341, P > 0.05).

### Frequency of enterococci in rural and urban patients

There was high isolation of both *E. faecalis* and *E. faecium* from urban patients than the rural patients (Table 6). There were 08/09 (88.9%) VRE from urban patients and 01/09 (9.1%) from rural. In 29 nasal isolates, all the 03/03 VRE (02 *E. faecalis* and 01 *E. faecium*) were from urban patients. VSE from urban and rural were 18/26 (26.9% *E. faecalis* and 42.3% *E. faecium*) and 08/26 (7.7% *E. faecalis* and 23.1% *E. faecium*) respectively. However, no statistically significant association of residential status with nasal isolates was present (Chi-square test: 5.569, P > 0.05).

From 56 perirectal isolates, VRE were 05/06 (83.3%) from urban including 03 *E. faecalis* and 02 *E. faecium* and 01/06 (16.7%) *E. faecium* from rural while VSE were 30/50 (34% *E. faecalis* and 22% *E. faecium*) from urban and 20/50 (18% *E. faecalis* and 22% *E. faecium*) from rural patients. Association was statistically not significant with perirectal isolates (Chi-square test: 4.249, P > 0.05).

### Association between clinical diagnosis and enterococcal colonization

The admitted patients with different disease conditions were categories into seven groups that were aspiration pneumonia, meningitis, pneumonia, renal failure, tetanus, tuberculosis and miscellaneous group. Diseases which appeared in more than five patients were given a separate group and diseases which were in less than five patients were grouped into “miscellaneous group”. Patients presented with pneumonia and with miscellaneous diseases were more colonized with VRE and VSE. In nasal VSE, 20/26 (26.9% *E. faecalis* and 50% *E. faecium*) isolates were from pneumonia patients, while 01/26 (3.9% *E. faecium*) isolate was from a patient with meningitis and 05/26 (7.7% *E. faecalis* and 11.5% *E. faecium*) were from the miscellaneous group. There were 02/03 VRE (*E. faecalis*) from pneumonia and 01/03 VRE (*E. faecium*) was from a patient in miscellaneous group (Table 6). However, no significant association was found with nasal isolates (Chi-square test: 9.350, P > 0.05).

Similarly, in perirectal VSE, 29/50 (30% *E. faecalis* and 28% *E. faecium*) were isolated from pneumonia patients followed by other leading group “miscellaneous group” which were having 16/50 (14% *E. faecalis* and 18% *E. faecium*) enterococci. Remaining 04/50 isolates (6% *E. faecalis* and 2% *E. faecium*) were from patients with meningitis. No significant association with perirectal isolates (Chi-square test: 22.958, P > 0.05). There were 12 patients who were suffering with pneumonia harboring enterococci both in nasal and perirectal samples. There was no significant correlation with other parameters of clinical data.

## Discussion

*E. faecalis* and *E. faecium* are potentially good focal species for microbiological surveillance study as they accounts for 80 to 90% of human enterococcal infections [17]. These two species were focused in the present study also because these are the common nosocomial agents and normal inhabitants of human intestinal tract, female genital tract, and less commonly in oral cavity [18-20]. *E. faecalis* is the most frequently occurring species of enterococci [21] than others but in the present study, results depict that the isolation rates of both species *E. faecalis* and *E. faecium* were almost equal. Isolation of VRE is highly significant both in causing infection in the individuals themselves and in transmission of vancomycin resistance to staphylococci. In the present study, isolation of VRE and colonization of VSE in nasal samples is alarming although the carriage rate of nasal and perirectal VRE is not much high. A study by Karimi et al. [22] 16.9% VRE were isolated from stool samples of hospitalized children. This isolation rate is much higher as compared to the present study. Burger & Muller [23] reported the carriage rate of glycopeptide resistant enterococci (GRE) from different body sites. They concluded that in 20 patients, the GRE isolation was most frequently from stool samples (95%) whereas from other sites, including mouth, nose, throat, rectum and perineum recovery was low (25%). However, VRE isolation rate is very low in the present study but as a whole, there is high perirectal carriage rate of enterococci, which is usual. The nasal carriage rate is comparatively high in the present study, although the main areas of colonization and isolation for enterococci are stool, rectum and perirectal area [24]. Only few patients were positive for both perirectal-VRE and nasal-VRE. VRE were low in frequency but comparatively high in nasal samples. The higher nasal VSE colonization may be due to poor hygiene of the patients.

Out of several different genes mediating vancomycin resistance, vanA and vanB resistance gene were targeted for identification as these gene clusters can be acquired and often transferable [25]. PCR analysis successfully identified *E. faecium* and *E. faecalis* along with nasal and perirectal VRE using *ddl* and *vanA* and *vanB* primers respectively (Table 2) like study of Dutka-Malen et al. [26].

High antimicrobial resistance is a characteristic of the enterococci, although some species like *E. faecium* are intrinsically more resistant than others [27]. In the present study, both the nasal and perirectal *E. faecium* isolates were more resistant than *E. faecalis* isolates. None of the nasal and perirectal isolates including VRE were resistant to linezolid and quinupristin/dalfopristin, the drugs of choice for these isolates. All the isolates showed high resistance to gentamicin like a previous study [28]. In an Iranian study [29] MICs of vancomycin for VRE isolates were from 32 to 512 μg/ml with similar results in our study. MICs of tetracycline for nasal and perirectal enterococci were from 2 to 256 mg/L and 0.5 to 256 mg/L respectively, which correspond with another report [30]. Resistance to ciprofloxacin was higher than other reports [31].

The urban patients were more colonized with VRE than the rural patients. This difference might be due to the irrational use of antibiotics in urban community. In a study by Oberoi & Aggarwal [32] high frequency of *E. faecium* in urban hospitalized patients was observed and that could be due to chronicity of cases or wider use of broad-spectrum antibiotics.

In a study by Berk & Verghese [33] reported some Gram-positive cocci including enterococci have significance in nosocomial respiratory infection and there are chances of occurrence of enterococcal pneumonia in patients receiving broad-spectrum antibiotics [34,35]. In the present study, there is more isolation of enterococci both *E. faecalis* and *E. faecium*, from the patients of pneumonia. This might have some correlation with nosocomial respiratory infection. More prospective study in this regard is under consideration. Isolation rate of VRE was low and is not possible to correlate it with vancomycin use as has been reported that treatment with vancomycin is not a risk factor for VRE colonization and infection [36,37]. The present study was only a microbiological surveillance study and not a study of intervention to decrease colonization rate and analysis of nosocomial infections caused by VRE.

## Conclusion

High nasal and perirectal colonization rate by *E. faecalis* and *E. faecium* in children in PICUs in particular 2.7% VRE and 23.6% VSE nasal colonization is alarming as the anterior nares are not the common niche for these. Further studies are required to elaborate transfer of vancomycin-resistance genes in Staphylococcal nasal carriers co-colonized with VRE.

## Abbreviations

BAA: Bile aesculin agar; bp: Break point; *E. faecalis*: *Enterococcus faecalis*; *E. faecium*: *Enterococcus faecium*; GRE: Glycopeptide resistant enterococci; KFSA: KF streptococci agar; MHA: Mueller Hinton Agar; MICs: Minimum inhibitory concentrations; *S. aureus*: *Staphylococcus aureus*; SBA: Sheep blood agar; SPSS: Statistical package for social sciences; PICU: Paediatrics intensive care unit; VRE: Vancomycin-resistant enterococci; VSE: Vancomycin sensitive enterococci.

## Competing interests

The authors declare that they have no competing interests.

## Authors’ contribution

MAY and SI collected the clinical samples and performed the experiments; NA designed and supervised the whole study as well as edited the manuscript; MAY, AM and SAK analysed the clinical data and wrote the manuscript. All authors read and approved the final manuscript.

## Pre-publication history

The pre-publication history for this paper can be accessed here:

http://www.biomedcentral.com/1471-2334/13/156/prepub
